# Exploring the Mechanism of Tannic Acid Against *Pichia kudriavzevii* in the VK2/E6E7 Vaginal Epithelial Cell Line and Its Synergy with Azoles on Drug-Resistant *Candida* Species

**DOI:** 10.3390/pathogens15050464

**Published:** 2026-04-24

**Authors:** Meng Zhou, Kun Ren, Huan Mei, Hang Yang, Dongmei Li, Weida Liu, Lulu Zhang, Xiaodong She

**Affiliations:** 1 Department of Medical Mycology, Hospital for Skin Diseases, Institute of Dermatology, Chinese Academy of Medical Sciences & Peking Union Medical College, Nanjing 210042, China; 2 Jiangsu Provincial Key Laboratory of Dermatology, Nanjing 210042, China; 3 Centers for Pharmaceutical Preparations, Hospital for Skin Diseases, Institute of Dermatology, Chinese Academy of Medical Sciences & Peking Union Medical College, Nanjing 210042, China; 4 Department of Microbiology & Immunology, Georgetown University Medical Center, Washington, DC 20007, USA; 5 Center for Global Health, School of Public Health, Nanjing Medical University, Nanjing 210008, China; 6 Department of Dermatology, Jiangsu Province Hospital of Traditional Chinese Medicine, No. 155 Hanzhong Road, Qinhuai District, Nanjing 210029, China

**Keywords:** *Pichia kudriavzevii*, VVC, tannic acid, vaginal epithelial cells, azoles resistance, *Candida* species

## Abstract

Vulvovaginal candidiasis (VVC) is a common gynecological infection, with *Pichia kudriavzevii* emerging as a significant pathogen due to its intrinsic fluconazole resistance and biofilm-forming capacity. This study investigates the antifungal efficacy and mechanisms of tannic acid (TA) against *P. kudriavzevii*, as well as its potential to reverse azole resistance across multiple Candida species with distinct resistance profiles. TA significantly inhibited *P. kudriavzevii* growth, surface colonization, and virulence gene expression at 3 μg/mL. Mechanistically, TA protected the human vaginal epithelial cell line VK2/E6E7 by reducing ROS levels, restoring mitochondrial membrane potential, and suppressing IL-1β and IL-18 release through modulation of the NLRP3-Caspase1-ASC axis. Furthermore, TA demonstrated synergistic activity when combined with azoles against five clinically azole-resistant *Candida* isolates spanning three *Candida* species with distinct resistance mechanisms: *P. kudriavzevii* (intrinsic), *C. albicans* (acquired), and *N. glabrata* (FKS-mediated). This study highlights TA as a promising natural therapeutic agent for *P. kudriavzevii* infections and offers a novel strategy for combating multidrug-resistant *Candida* through combination therapy.

## 1. Introduction

Fungal infections have become a pressing global concern, with invasive candidiasis and candidemia posing a substantial public health problem due to their alarming mortality rates [1,2,3]. *Pichia kudriavzevii*, alternatively known as *Candida krusei*, has emerged as a significant and emerging pathogen, accounting for approximately 1.5% to 8% of candidemia cases [4,5]. *P. kudriavzevii*, classified within the order Saccharomycetales and the Agenus Candida, is capable of causing a wide range of infections, including cutaneous infections, candidemia, and infections of visceral organs [6]. Additionally, as a facultative anaerobe that can produce pseudohyphae, *P. kudriavzevii* possesses a thick cell wall and an abundance of surface glycoproteins, which endow it with strong adhesion capabilities within the host [7]. Unfortunately, *P. kudriavzevii* exhibits intrinsic resistance and reduced susceptibility to azoles and polyenes, two major classes of antifungal agents [5]. In recent years, although echinocandins have been regarded as a good therapeutic option for the treatment of invasive *P. kudriavzevii* infections, studies have demonstrated the rapid acquisition of resistance by *P. kudriavzevii* during treatment with caspofungin [8]. Therefore, there is an urgent need to discover novel antifungal agents to replace existing therapies [9].

Vulvovaginal candidiasis (VVC) is a common gynecological condition caused by the overgrowth of *Candida* species, with approximately 75% of women experiencing at least one episode in their lifetime [10]. *Candida albicans* (*C. albicans*) is the predominant pathogen responsible for VVC, accounting for 80% to 95% of all cases worldwide [11]. However, in recent years, there has been an increase in VVC cases caused by non- *C. albicans* species. *P. kudriavzevii*, although less common, is an important pathogen in VVC, representing about 1% of cases and exhibiting intrinsic resistance to fluconazole, a frequently used antifungal agent [12,13]. Additionally, *P. kudriavzevii* is more frequently associated with recurrent VVC and is more prevalent in older women [13]. Critically, VVC involves multiple Candida species with distinct resistance mechanisms. *P. kudriavzevii* exhibits intrinsic azole resistance, whereas *Candida albicans* (*C. albicans*) typically acquires resistance through target site mutations and efflux pump overexpression, and *Nakaseomyces glabrata* (*N. glabrata*) frequently demonstrates echinocandin resistance via FKS mutations [14,15,16,17]. This species-specific diversity in drug resistance poses a significant clinical challenge, as effective adjuvant therapies must demonstrate efficacy across the spectrum of VVC pathogens rather than target a single species. Consequently, validation of novel therapeutic agents against multiple clinically relevant *Candida* species is essential for developing broadly applicable treatment strategies. Therefore, there is an urgent need to develop new therapeutic agents targeting *P. kudriavzevii* to address the clinical challenges in treating VVC.

Tannins, a group of naturally occurring polyphenol compounds, are widely present in various tree species and higher plants [18]. In recent years, there has been growing attention towards the pharmacological efficacy of tannins [19]. TA, extracted from plant sources like gallnuts, is a hydrolysable gallic acid tannin with low toxicity. Numerous studies have demonstrated that TA exhibits diverse pharmacological activities, such as anticancer, anti-inflammatory, neuroprotective, antiviral and antioxidant effects [20,21]. Additionally, it is noteworthy that TA exerts antifungal effects, albeit with limited research in this area [19]. Here, we evaluated the inhibitory effect of TA on various *Candida* species, and the results showed that TA displayed extremely strong antifungal activity against *P. kudriavzevii* as well as synergistic chemosensitizing effects against fluconazole-resistant *C. albicans* and *N. glabrata* isolates, expanding the therapeutic range of TA.

## 2. Materials and Methods

### 2.1. Strains and Cells

The standard strain of *P. kudriavzevii*, C6d (ATCC 6258), was kindly provided by Peking University First Hospital. The clinical fluconazole-resistant *C. albicans* isolates (06-37, 09-07, C1-17, and C1-19) and the *N. glabrata* isolate (Y10-5) were obtained from our team’s previous multicenter epidemiological study on VVC in China [22]. The *Candida* strains were activated on yeast extract peptone dextrose (YPD) solid medium, and single colonies were selected and inoculated into liquid YPD medium for further cultivation. The human vaginal epithelial cell line VK2/E6E7 (ATCC CRL-2616) was cultured in Keratinocyte-SFM (Gibco, Grand Island, NY, USA).

### 2.2. MTS Assay

MTS (3-(4,5-dimethylthiazol-2-yl)-5-(3-carboxymethoxyphenyl)-2-(4-sulfophenyl)-2H-tetrazolium) assay was used to assess the viability of *P. kudriavzevii* after TA treatment.

To evaluate TA’s inhibitory effect on *P. kudriavzevii* surface colonization, we modified the CLSI M27-A3 broth microdilution reference method (CLSI, 2008) as follows: a fixed inoculum of 3 × 10^3^ CFU/mL was used with selected TA concentrations (0, 1, 3, 9 μg/mL) based on preliminary cytotoxicity screening; and cell viability was quantified using the MTS colorimetric assay rather than visual turbidity assessment to enable objective, high-throughput detection. Firstly, *P. kudriavzevii* inocula were standardized to 3 × 10^3^ CFU/mL in RPMI 1640 medium supplemented with L-glutamine and buffered with 0.165 M MOPS (pH 7.0 ± 0.1), then seeded into 96-well plates (100 μL/well) and treated with TA at indicated concentrations (0, 1, 3, 9 μg/mL), followed by incubation at 35 °C for 48 h. Subsequently, 20 μL CellTiter 96^®^ Aqueous solution reagent (Promega, Madison, WI, USA) was added into each well and incubated for 1 h at 30 °C. Finally, the absorbance of each well was detected at 490 nm using MultiskanGO (Thermo Scientific, Waltham, MA, USA). The modified method was validated by correlation with CFU enumeration and LIVE/DEAD staining.

### 2.3. Candida Proliferation Assay

After growing the test strain on YPD solid medium for 48 h, 3 to 5 colonies were selected and used to prepare a suspension with a concentration of 3 × 10^3^ CFU/mL in RPMI 1640 medium. This suspension was then inoculated into a 96-well cell culture plate (200 μL/well), and the plate was placed into a real-time label-free cell dynamic analyzer (Nanoanalytics, Nordrhein-Westfalen, Germany), where data acquisition was performed every 6 h. The total duration for monitoring the growth dynamics of the test strain was 48 h.

### 2.4. Hyphal Formation Assay

Before experiments, *P. kudriavzevii* was grown in 30 mL YPD broth and incubated overnight at 30 °C. RPMI1640 medium containing 10% FBS was prepared. Subsequently, the *P. kudriavzevii* strains were cultured in RPMI1640 medium with or without TA at a density of 1 × 10^7^ cells/mL. They were placed on a shaker (200 rpm) and incubated for 24 h at 30 °C. Finally, the effect of TA on the hyphal formation of *P. kudriavzevii* was observed under optical microscope (Leica Microsystems, Wetzlar, Germany).

### 2.5. Adhesion Assay

To evaluate TA’s inhibitory effect on *P. kudriavzevii* surface colonization, we modified the CLSI M27-A3 broth microdilution method as follows: Initially, poly-l-lysine-coated glass coverslips were placed in a 12-well cell culture plate, and 1 mL of *P. kudriavzevii* suspension (1 × 10^6^ CFU/mL) was added to each well. Subsequently, biofilms were formed by incubating the *P. kudriavzevii* at 30 °C for 24 h in the presence of various concentrations of TA. The biofilms not treated with TA served as the live controls, while those treated with isopropanol for 60 min were used as dead controls. The formed biofilms were then subjected to immunofluorescence staining using the LIVE/DEAD FungaLight Yeast Viability Kit (Molecular Probes, Inc., Eugene, OR, USA). Live yeasts with intact cell membranes were stained fluorescent green by SYTO9, whereas dead yeasts with damaged membranes were stained fluorescent red, indicating the penetration of propidium iodide. According to the manufacturer’s protocol, 500 µL of staining solution was added to each well and incubated in the dark at 30 °C for 30 min. The biofilms were observed and photographed under a confocal laser scanning microscope at 10× magnification.

### 2.6. Lactate Dehydrogenase (LDH) Assay

The toxic effects of *P. kudriavzevii* induction on vaginal epithelial cells were assessed by measuring LDH release in VK2 cells. Firstly, VK2 cells were seeded uniformly in 96-well plates (1 × 10^5^ cells/well). Subsequently, *P. kudriavzevii* (1 × 10^5^ cells/well) or TA was added to 96-well plates and the incubation continued for 6, 12, 24, or 48 h. Then, the LDH detection reagent (Beyotime, Beijing, China) was added to each well and reacted at 37 °C for 1 h. Finally, the plate was placed in microplate reader (Thermo MultiskanGO, Waltham, MA, USA) to detect the absorption value at 490 nm.

### 2.7. qRT-PCR Experiment

The total RNA of *P. kudriavzevii* was extracted using TRizol reagent (#15596026, Invitrogen, Thermo Fisher Scientific, Carlsbad, CA, USA) and was co-incubated with DNase I (10 µL/1 U) at 37 °C for 1 h to eliminate potential DNA contamination. Subsequently, the RNA was reverse-transcribed into cDNA using the Evo M-MLV RT Kit (Agbio, AG11728, Changsha, China). Finally, qRT-PCR analysis was performed using SYBR Green Pro Taq HS Mix (Agbio, AG11701, Changsha, China). The primers used in this study are summarized in Supplementary Table S1. Data normalization was performed using two independent reference genes, ACT1 and 18S ribosomal RNA, to ensure the reliability and robustness of our quantitative results. The relative gene expression levels were calculated using the ΔΔCt method.

### 2.8. Reactive Oxygen Species (ROS) Detection Assay

The release of ROS in VK2 cells was detected using a commercial kit. Initially, VK2 cells were seeded into 6-well plates (1 × 10^6^ cells/well). Subsequently, a suspension of *P. kudriavzevii* (2 × 10^6^ CFU/well) was added to the wells. Additionally, either blank solvent or TA solution was introduced into the wells. After 48 h, the cells were labeled with DCFH-DA (Beyotime, Beijing, China) according to the manufacturer’s instructions. Finally, the cells were observed and photographed under a fluorescence microscope (Leica Microsystems, Wetzlar, Germany).

### 2.9. Mitochondrial Membrane Potential Detection Assay

The mitochondrial membrane potential (MMP) in VK2 cells was assessed using a Mitochondrial Membrane Potential Assay Kit (Beyotime, Beijing, China) with JC-1. VK2 cells were seeded into 6-well plates (1 × 10^6^ cells/well) and incubated for 1 h to allow cell attachment. Subsequently, a suspension of *P. kudriavzevii* (2 × 10^6^ CFU/well) was added to the wells. Additionally, either blank solvent or TA solution was introduced into the wells. After 48 h, cells were stained with JC-1 according to the kit instructions (37 °C, dark, 30 min), followed by washing. The fluorescence intensity of JC-1 monomers (green) and aggregates (red) was detected using a confocal laser scanning microscope (Carl Zeiss AG, Oberkochen, Germany). The MMP was evaluated by analyzing the red-to-green fluorescence ratio.

### 2.10. Fluorescein Isothiocyanate (FITC) and PKH26 Fluorescence Staining Assay

*P. kudriavzevii* suspensions were irradiated with 254 nm UV light for 45 min and then incubated with 1.25 mM FITC at 4 °C overnight in the dark. After centrifugation and three PBS washes, FITC-labeled *P. kudriavzevii* were resuspended in SFM medium. VK2 cells were digested with trypsin, collected by centrifugation, washed with PBS, and then incubated with 4 μM PKH-26 (Sigma-Aldrich, St. Louis, MO, USA) in Solution C for 5 min. After adding an equal volume of serum and incubating for 1 min, VK2 cells were washed three times with medium to obtain PKH-26-labeled cells. FITC-labeled *P. kudriavzevii* were co-cultured with PKH-26-labeled VK2 cells at an MOI of 5. The co-culture was treated with 3 μg/mL TA or blank solvent for 48 h. Images were captured using a confocal laser scanning microscope with FITC (Ex/Em = 490/530 nm) and PKH-26 (Ex/Em = 551/585 nm) channels.

### 2.11. Immunofluorescence Assay

VK2 cells were seeded onto cell culture slides in 12-well plates. Subsequently, 5-fold excess of *P. kudriavzevii* or 3 µg/mL TA was added and incubated for 6 h. Cells were washed thrice with PBS and fixed with 4% paraformaldehyde for 15 min, followed by three PBS washes. Cells were permeabilized with 0.1% Triton-100 in PBS for 10 min on ice, washed twice with PBS, and blocked with 5% BSA at room temperature for 1 h. Primary anti-NLRP3 antibody (CST, #15101, 1:500) was applied and incubated overnight at 4 °C. After PBST washing, cells were incubated with the corresponding fluorescent secondary antibody for 1 h in the dark, followed by DAPI staining of nuclei. Fluorescence images were captured using a confocal laser scanning microscope.

### 2.12. Western Blotting Assay

Proteins were extracted using RIPA lysis buffer (Beyotime, Beijing, China) containing protease inhibitors (Thermo Fisher, Waltham, MA, USA) and quantified by BCA protein assay kit (Thermo Fisher Scientific, Waltham, MA, USA). Total protein extracts were separated via SDS-PAGE and transferred onto polyvinylidene fluoride membranes. The membranes were blocked in 5% BSA and incubated overnight at 4 °C with primary antibodies against NOD-like receptor family pyrin domain containing 3 (NLRP3) (Proteintech, Wuhan, China, #15101), Apoptosis-associated speck-like protein containing a CARD (ASC) (Proteintech, #10500), Cysteinyl Aspartate Specific Proteinase1 (Caspase1) (Proteintech, Wuhan, China, #22915), and Glyceraldehyde-3-phosphate dehydrogenase (GAPDH) (Engibody, Shanghai, China, AT0002). Subsequently, membranes were incubated for 1 h at room temperature with horseradish peroxidase-conjugated secondary antibody (anti-rabbit; 1:3000). Target protein bands were detected using an ECL detection system (Thermo Fisher Scientific, Waltham, MA, USA).

### 2.13. Broth Microdilution Assay

To evaluate the interaction between tannic acid (TA) and fluconazole, the checkerboard broth microdilution assay was performed according to the CLSI M27-A3 guidelines with minor modifications. Briefly, TA and fluconazole were dissolved in Mueller–Hinton Broth (MHB) and subjected to 2-fold serial dilutions, yielding final concentration ranges of 1.5–24 µg/mL for TA and 0.25–256 µg/mL for fluconazole. Fungal suspensions from 24-h cultures were added to each combination of drug concentrations to achieve a final inoculum density of 1–5 × 10^5^ CFU/mL. The inoculated 96-well flat-bottom microtiter plates (Corning, NY, USA) were incubated at 37 °C for 48 h. Candida growth was assessed by visual inspection of turbidity. Each test was performed in triplicate. Drug interactions were quantified using the Fractional Inhibitory Concentration (FIC) index, calculated as FIC = [MIC (TA in combination)/MIC (TA alone)] + [MIC (fluconazole in combination)/MIC (fluconazole alone)]. This formula enables systematic evaluation of the synergistic inhibitory effect by comparing the minimum inhibitory concentrations (MICs) of each agent when used in combination versus alone. Mean FIC values from three independent experiments were used for final interpretation. Drug interactions were interpreted as follows: FICI ≤ 0.5 indicated synergy, 0.5 < FICI ≤ 1.0 indicated additivity, 1.0 < FICI < 4.0 indicated indifference, and FICI ≥ 4.0 indicated antagonism.

### 2.14. Statistical Analysis

Statistical analyses were performed using GraphPad Prism 8.0 software (GraphPad Software, San Diego, CA, USA). All experiments included at least three independent biological replicates. Data distribution was assessed for normality using the Shapiro–Wilk test. Normally distributed data are presented as mean ± standard deviation (SD) and were analyzed by unpaired Student’s *t*-test for two-group comparisons or one-way ANOVA followed by Tukey’s post hoc test for multi-group comparisons (≥3 groups). Non-normally distributed data are presented as median (IQR) and were analyzed using Mann–Whitney U test or Kruskal–Wallis test with Dunn’s post hoc test. A *p*-value < 0.05 was considered statistically significant.

## 3. Results

### 3.1. TA Exhibited a Significant Inhibitory Effect on the Growth of P. kudriavzevii

**Figure 1:**
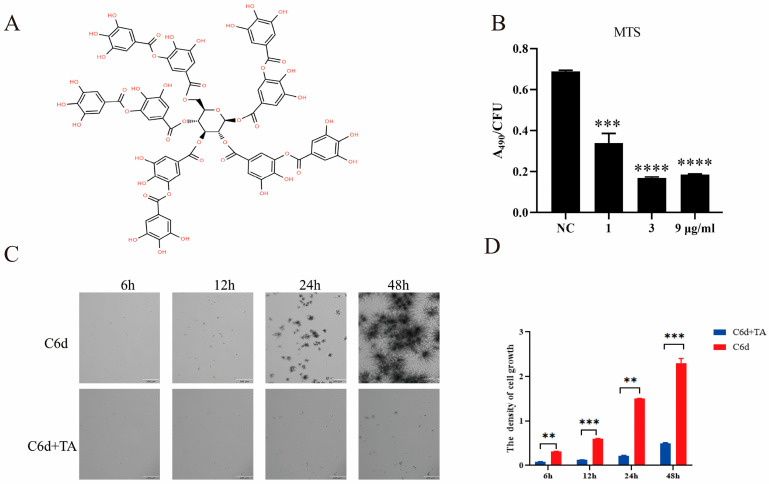
TA significantly suppresses the growth of *P. kudriavzevii*. (**A**) The chemical structure of TA. (**B**) MTS assays were performed to assess the changes in proliferation viability of *P. kudriavzevii* following treatment with different concentrations of TA for 24 h. (**C**,**D**) TA at concentrations of 1, 3, and 9 μg/mL markedly inhibited the proliferation of *P. kudriavzevii*. RTCA assay showed that TA distinctly inhibited the growth viability of *P. kudriavzevii* in a time-dependent manner. Bar = 200 μm. Statistics in (**B**) by one-way ANOVA with Dunnett’s multiple comparisons test; statistics in (**D**) by two-way ANOVA followed by Sidak’s multiple comparisons test; data are shown as mean ± s.d. ** *p* < 0.01, *** *p* < 0.001, **** *p* < 0.0001.

Firstly, we explored whether TA exerted potential inhibitory effects on *P. kudriavzevii*. The MTS assays were performed to assess the changes in proliferation viability of *P. kudriavzevii* following treatment with different concentrations of TA for 24 h. As shown in Figure 1B, TA at concentrations of 1, 3, and 9 μg/mL markedly inhibited the proliferation of *P. kudriavzevii*. Considering that the inhibitory effect of 3 μg/mL TA on *P. kudriavzevii* was the strongest, this concentration was selected for further investigation. Additionally, RTCA (Real-Time Cell Analysis) assay showed that TA distinctly inhibited the growth viability of *P. kudriavzevii* in a time-dependent manner (Figure 1C,D). Together, these results revealed that TA demonstrated potent antifungal efficacy against *P. kudriavzevii*.

### 3.2. TA Dramatically Inhibited the Hyphal Formation of P. kudriavzevii

**Figure 2:**
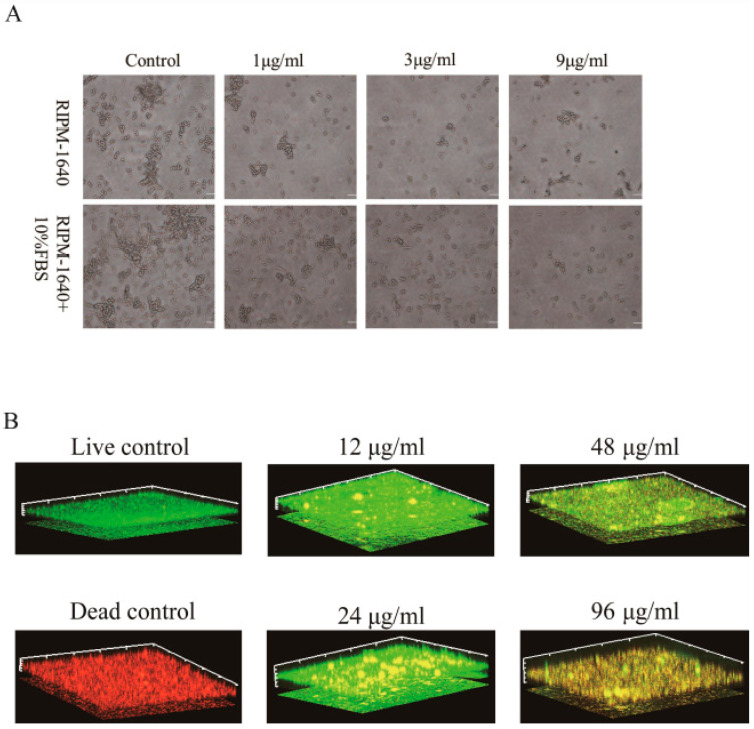
TA obviously suppresses hyphal formation in *P. kudriavzevii* and notably disrupts the viability and spatial integrity of the *P. kudriavzevii* adhesion in a concentration-dependent manner. (**A**) *P. kudriavzevii* was cultured at 37 °C in RPMI-1640 medium (upper figure) and RPMI-1640 medium supplemented with 10% fetal bovine serum (FBS) (lower figure). Specified concentrations of TA were applied to *P. kudriavzevii* for 24 h. After incubation, samples were retrieved and photographed under a 40× objective lens. Bar = 20 μm. (**B**) *P. kudriavzevii* was cultured at 35 °C in a 12-well cell culture plate, and specific concentrations of TA were applied to the fungus for 24 h. Biofilms were formed on poly-L-lysine-coated glass coverslips. The adhesion viability was assessed using SYTO 9 (green fluorescence) and PI (red fluorescence) staining. Yellow fluorescence indicates the overlap of green and red signals, representing areas with both live and dead cells.

Hyphal is considered one of the most virulent factors of *Candida*. Inhibition of hyphal formation was expected to be one of the effective strategies for screening new antifungal drugs [23]. Therefore, to further validate the inhibitory effect of TA on *P. kudriavzevii*, we utilized optical microscopy to observe the impact of different concentrations of TA on the hyphal formation of *P. kudriavzevii* after 24 h of exposure. As shown in Figure 2A, TA obviously suppressed hyphal formation in *P. kudriavzevii* in a concentration-dependent manner.

### 3.3. TA Significantly Inhibited P. kudriavzevii Surface Colonization and Disrupted Microcolony Architecture

*P. kudriavzevii* has greater cell hydrophobicity than other *Candida* species, making it easy to colonize host cells and rapidly multiply to form a dense network of biofilms, which reduces its sensitivity and even produces resistance to antifungal drugs [24,25,26]. Here, the effect of TA on *P. kudriavzevii* biofilms was explored by using confocal laser scanning microscopy (CLSM). As shown in Figure 2B, the adhesion of normal *P. kudriavzevii* presented a regular and compact three-dimensional structure, but TA dramatically destroyed the normal surface colonization structure of *P. kudriavzevii*. In addition, with the increase in TA concentration, the structure of *P. kudriavzevii* surface colonization was damaged more severely and the number of *P. kudriavzevii* cells decreased more sharply (Figure 2B). In brief, TA prominently inhibited *P. kudriavzevii* adhesion and damaged the surface colonization.

### 3.4. TA Markedly Down-Regulated the Expression of Virulence-Related Genes in P. kudriavzevii

**Figure 3:**
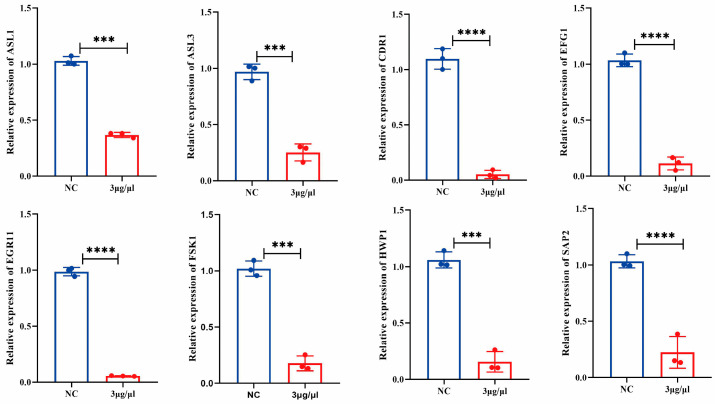
TA markedly down-regulated the expression of virulence-related genes in *P. kudriavzevii*. The expression levels of virulence-associated genes (ASL1, ASL3, CDR1, EFG1, EGR11, FSK1, HWP1, and SAP2) in *P. kudriavzevii* were quantified using qRT-PCR analysis. All gene expression values shown are ACT1-normalized relative quantities. Two-tailed unpaired *t*-tests were performed for each gene independently; data are shown as mean ± SD from three independent experiments. *** *p* < 0.001, **** *p* < 0.0001.

To further explore the mechanism by which TA inhibited *P. kudriavzevii*, qRT-PCR analysis was performed to evaluate the effect of TA on the expression levels of virulence-related genes. It has been reported that EFG1 [27,28,29], agglutinin-like sequence (ALS) [30,31] and hyphal wall protein 1 (Hwp1) [32] are closely related to hyphal formation and adhesion in *Candida* spp. Here, our data showed that TA significantly inhibited the mRNA expression levels of EFG1, ALS1, ALS3, and Hwp1 (Figure 3). Furthermore, TA obviously inhibited the expression of targets associated with fungal resistance, such as CDR1 and 1,3-beta-D-glucan synthase (FKS1). Not only that, but TA also remarkably inhibited the expression of secreted aspartic protease 2 (SAP2), which were proven to be a crucial virulent factor during fungal infections [33]. The target genes were selected based on their established roles in distinct virulence mechanisms: EFG1 (master regulator of hyphal morphogenesis) [28,34,35,36], ALS1/3 (adhesion and biofilm formation) [28,37,38], HWP1 (hyphal-specific host adhesion) [39,40], CDR1 (drug efflux and resistance) [41,42], FKS1 (cell wall integrity) [43,44], and SAP2 (tissue invasion and nutrient acquisition) [33,45].

### 3.5. TA Alleviated the Damage of P. kudriavzevii on Human Vaginal Epithelial Cells

**Figure 4:**
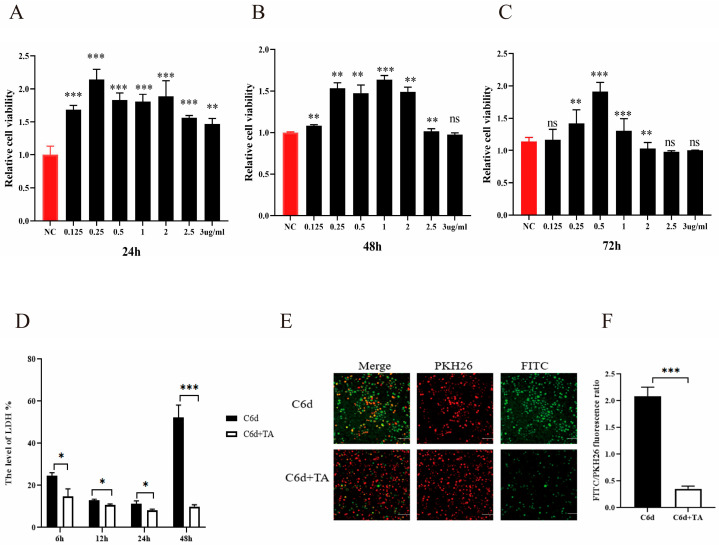
TA alleviated the damage of *P. kudriavzevii* on human vaginal epithelial cells. (**A**–**C**) CCK8 assays were performed to assess the changes in VK2 cells viability treatment with different concentrations of TA for 24 h, 48 h and 72 h. (**D**) The percentage of vaginal epithelial cell damage induced by *P. kudriavzevii* in the control group and the group treated with 3 μg/mL TA was assessed by measuring the release of LDH at various time points. (**E**) FITC was used to stain *P. kudriavzevii* (green), while PKH26 was used to stain VK2 cells (red). Blank solvent or 3 μg/mL TA was added to the co-culture system of *P. kudriavzevii* and vaginal epithelial cells. After 48 h, images were captured using a confocal laser scanning microscope. (**F**) Quantitative analysis of FITC and PKH26 fluorescence signals was performed for (**E**). Statistics in (**A**–**C**) were performed using one-way ANOVA with Dunnett’s multiple comparisons test. Statistics in (**D**) were performed using a 2-way ANOVA followed by Sidak’s multiple comparisons test. Statistics in (**F**) were performed using a two-tailed unpaired *t*-test; data are shown as mean ± s.d. * *p* < 0.05, ** *p* < 0.01, *** *p* < 0.001; ns, not significant.

To explore the therapeutic effect of TA on VVC, we first investigated the effect of TA on the activity of human vaginal epithelial cells by Cell Counting Kit-8 (CCK8) assay. As shown in Figure 4A–C, the viability of human vaginal epithelial cells was significantly enhanced after treatment with TA (0.125~2.5 μg/mL) for 24 h or 48 h, respectively. However, 3 μg/mL TA had no obvious effect on the activity of human vaginal epithelial cells, so we chose 3 μg/mL TA to evaluate whether TA alleviated the damage of human vaginal epithelial cells induced by *P. kudriavzevii*. Subsequently, LDH assay showed that *P. kudriavzevii* markedly induced the damage of human vaginal epithelial cells in a time-dependent manner, while 3 μg/mL TA could significantly reverse this effect (Figure 4D). Additionally, we observed a similar phenomenon by confocal fluorescence microscopy (Figure 4E,F).

### 3.6. TA Significantly Decreased the ROS Levels in Human Vaginal Epithelial Cells Induced by P. kudriavzevii

**Figure 5:**
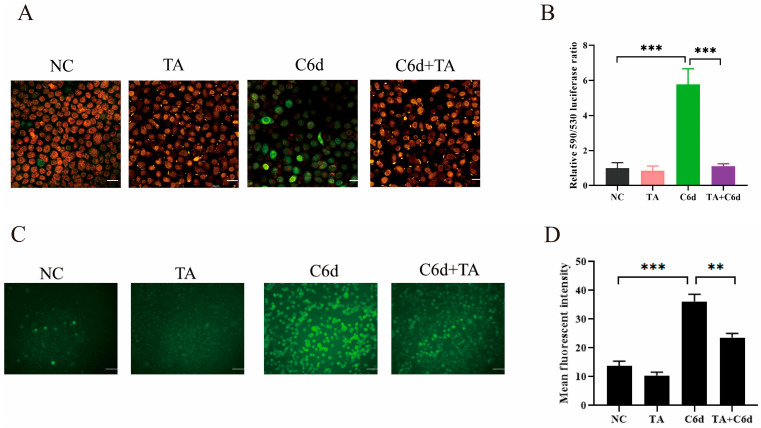
TA significantly ameliorated MMP and the ROS levels in human vaginal epithelial cells induced by *P. kudriavzevii.* (**A**,**B**) Under the specified conditions, VK2 cells were stained with JC-1 and captured using a confocal microscope. Bar = 30 μm. (**A**). The fluorescence intensities at 590 nm (green) and 530 nm (red) were quantitatively analyzed (**B**). (**C**,**D**) Under the specified conditions, the intracellular ROS levels were measured using the DCFH-DA kit, and images were captured using a fluorescence microscope. Bar = 50 μm (**C**). The fluorescence intensity was then quantitatively analyzed (**D**). Statistics in (**B**,**C**) were performed using one-way ANOVA with Tukey’s multiple comparisons test; data are shown as mean ± s.d. ** *p* < 0.01, *** *p* < 0.001.

Mitochondrial dysfunction leads to a decrease in mitochondrial membrane potential (MMP) and the accumulation of reactive oxygen species (ROS). TA contains many phenolic hydroxyl groups in its molecular structure (Figure 1A), which has strong reducibility. Therefore, we attempted to explore the effect of TA on ROS levels in human vaginal epithelial cells. As shown in Figure 5A,B, ROS levels in human vaginal epithelial cells were significantly increased after infection with *P. kudriavzevii*. Interestingly, 3 μg/mL TA could significantly inhibit the ROS level of human vaginal epithelial cells induced by *P. kudriavzevii*. ROS are closely related to changes in MMP. Therefore, we subsequently detected the MMP in human vaginal epithelial cells using JC-1. As shown in Figure 5C,D, *P. kudriavzevii* induces significant depolarization in human vaginal epithelial cells, while TA (3 µg/mL) significantly alleviates the abnormal mitochondrial membrane potential induced by *P. kudriavzevii*.

### 3.7. TA Protected Human Vaginal Epithelial Cells Infected with P. kudriavzevii via the NLRP3 Inflammasome

**Figure 6:**
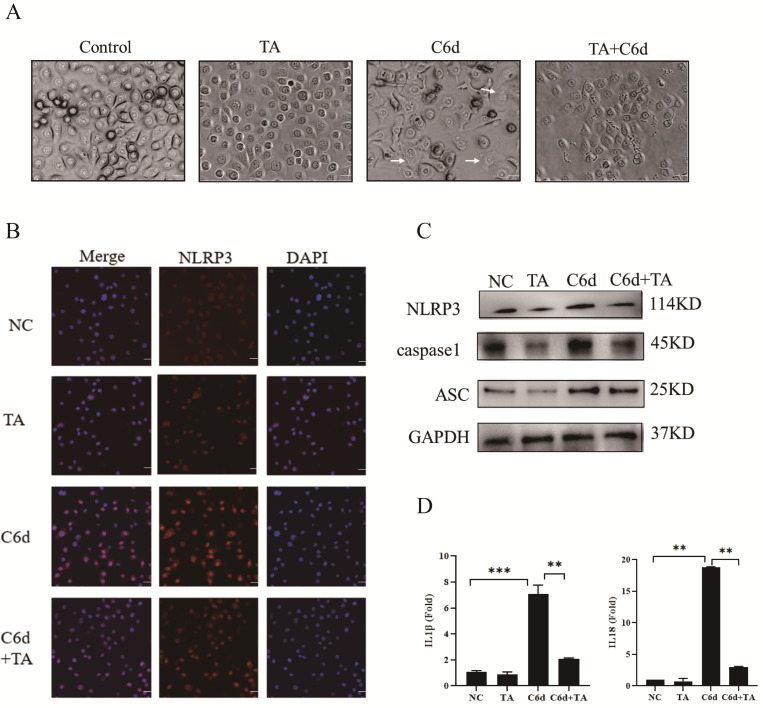
TA protects VK2 cells infected with *P. kudriavzevii* via the NLRP3 inflammasome. (**A**) Under different treatment conditions, the morphology of VK2 cells was observed under a microscope. Arrows indicate representative cells exhibiting balloon-like vesicles characteristic of pyroptosis. Bar = 20 µm. (**B**) The expression level of NLRP3 in VK2 cells was detected by immunofluorescence. Bar = 20 µm. DAPI (blue) and NLRP3 (red). (**C**) WB analysis of NLRP3, Caspase1, ASC and GAPDH expression in VK2 cells treated under indicated condition. (**D**). ELISA analysis of IL-1β and IL18 protein expression in VK2 cells treated under indicated condition. Statistics in (**D**) were performed using one-way ANOVA; ** *p* < 0.01, *** *p* < 0.001.

As reported, ROS serve as a key activator of the NLRP3 inflammasome, which can induce pyroptosis by facilitating the assembly of the NLRP3-ASC-caspase-1 multiprotein complex [46,47,48]. The typical morphological characteristics of pyroptosis include membrane blebbing, cell swelling, and eventual membrane rupture [49,50]. Our study demonstrated that *P. kudriavzevii* could induce a significant spherical swelling structure in human vaginal epithelial cells (indicated by arrows), while TA could markedly attenuate this change (Figure 6A). This indicates that TA could ameliorate pyroptosis of human vaginal epithelial cells induced by *P. kudriavzevii*. Furthermore, immunofluorescence assays revealed that TA could significantly inhibit the induction of NLRP3 protein fluorescence intensity in human vaginal epithelial cells by *P. kudriavzevii* (Figure 6B). Additionally, Western blot analysis showed that TA could significantly reverse the decreased expression levels of NLRP3, ASC, and caspase-1 proteins caused by *P. kudriavzevii* infection (Figure 6C). ELISA revealed that *P. kudriavzevii* induces elevated levels of IL-18 and IL-1β in human vaginal epithelial cells, whereas TA (3 µg/mL) significantly reduces the levels of IL-18 and IL-1β induced by *P. kudriavzevii* (Figure 6D). In summary, these results indicate that TA may inhibit pyroptosis and inflammatory responses induced by *P. kudriavzevii* via the NLRP3-ASC-caspase-1 axis.

### 3.8. Synergistic Antifungal Activity of TA and Fluconazole Against Clinical Fluconazole-Resistant C. albicans and N. glabrata

In addition to *P. kudriavzevii*, *C. albicans* and *N. glabrata* are also important causative agents of VVC. We collected four clinical fluconazole-resistant strains of *C. albicans* and one clinical fluconazole-resistant strain of *N. glabrata* to further investigate whether the combination of TA and fluconazole could reduce the minimal inhibitory concentrations (MICs) of these *Candida* species. As expected, fluconazole alone required a high concentration of up to 128 µg/mL to significantly inhibit these resistant strains (Table 1). Similarly, the antifungal efficacy of tannic acid in combination with fluconazole against fluconazole-resistant Candida clinical isolates was evaluated using the Fractional Inhibitory Concentration Index (FICI). Drug interactions were interpreted as follows: FICI ≤ 0.5 indicated synergy, 0.5 < FICI ≤ 1.0 indicated additivity, 1.0 < FICI < 4.0 indicated indifference, and FICI ≥ 4.0 indicated antagonism. Checkerboard assays revealed that the combination of tannic acid and fluconazole exhibited pronounced synergistic activity (FICI ≤ 0.5) across all five tested strains (Table 1). This result confirms the groundbreaking synergistic effect of the combination of tannic acid and fluconazole against clinical fluconazole-resistant *Candida* species, further indicating that TA holds promise as a potential candidate for the treatment of VVC.

## 4. Discussion

*P. kudriavzevii* is a pathogen of candidiasis and an emerging opportunistic yeast that can rapidly develop acquired resistance to multiple antifungal agents. Therefore, it is imperative to develop new antifungal agents against *P. kudriavzevii* to address the limitations of current clinical treatments. TA, a phenolic compound widely found in plants, possesses various medicinal properties. In terms of antifungal activity, TA has been shown to inhibit the growth of *C. albicans* [51]. Moreover, TA can suppress the growth and adhesiveness of *Candida tropicalis* [52]. Additionally, TA has been reported to exhibit broad-spectrum antifungal activity against human pathogenic fungi and yeasts [53]. However, the antifungal mechanisms of TA, especially against the clinically important opportunistic pathogen *P. kudriavzevii*, have not been fully elucidated. In this study, we demonstrated that TA significantly inhibits the growth, hyphal formation, surface colonization, and expression of virulence-related genes (such as EFG1, HWP1, and CDR1) in *P. kudriavzevii*. Therefore, our findings suggest that TA may serve as a promising candidate for the development of antifungal agents against *P. kudriavzevii*.

VVC is one of the most common invasive fungal infections, and its disruption of vaginal microenvironment homeostasis significantly exacerbates the health burden on female patients [54,55]. The majority of VVC patients exhibit pathological changes such as mucosal barrier damage, abnormal elevation of local inflammatory factors, and reduced abundance of Lactobacillus [56,57]. In VVC, *P. kudriavzevii*, although the third most common pathogenic fungus after *C. albicans* and *N. glabrata*, poses a significant challenge due to its intrinsic resistance to azole drugs like fluconazole, which significantly limits the efficacy of traditional azole therapies. Importantly, clinical VVC often involves mixed infections or sequential colonization by multiple Candida species, each with distinct drug resistance profiles. Our comparative findings demonstrate that TA not only exerts direct antifungal effects against *P. kudriavzevii* but also significantly enhances fluconazole sensitivity in resistant isolates of C. albicans and N. glabrata. TA exhibits broad-spectrum resistance-reversing activity against Candida species, indicating that TA-based combination therapy may provide a unified treatment strategy for polymicrobial VVC, eliminating the need for species-specific drug selection and thereby streamlining clinical management of drug-resistant infections. Our study revealed that TA significantly mitigates host cell damage by reducing the release of LDH from human vaginal epithelial cells infected with *P. kudriavzevii*, decreasing fungal adhesion, and inhibiting the overproduction of ROS. TA demonstrates significant cell type selectivity in its biological activities—it exhibits cytotoxicity and pro-apoptotic effects against various tumor cells (bladder cancer, osteosarcoma, glioma, lung cancer, colon cancer) [58,59,60,61], while showing pro-proliferative and cytoprotective effects on normal cells such as osteoblasts [59], neuronal cells [62], and vaginal epithelial cells in this study. Therefore, our study expands the potential applications of TA and may provide a new therapeutic option for VVC caused by *P. kudriavzevii* in clinical settings.

ROS are primarily generated during the mitochondrial electron transport chain (ETC) process [63,64]. The mitochondrial membrane potential (MMP) can restrict excessive ROS production, thereby reducing oxidative stress-induced cellular damage. The integrity of the mitochondrial inner membrane is crucial for maintaining the stability of MMP. Disruption of membrane integrity not only impairs mitochondrial energy metabolism but also activates key signaling pathways such as pyroptosis [65,66]. Our study demonstrated that after treatment with TA for 48 h, human vaginal epithelial cells infected with *P. kudriavzevii* exhibited significant recovery of MMP, thereby alleviating the cytotoxic damage caused by *P. kudriavzevii*. Additionally, TA significantly reduced the levels of ROS and LDH release in human vaginal epithelial cells induced by *P. kudriavzevii*. These results suggest that in human vaginal epithelial cells, TA may maintain mitochondrial functional homeostasis by inhibiting ROS production and stabilizing MMP, thereby resisting cell damage caused by *P. kudriavzevii* infection.

Pyroptosis is a form of programmed cell death characterized by cell swelling, rupture of the cell membrane, and the release of large amounts of inflammatory factors, thereby triggering a robust inflammatory response [49,50]. This study demonstrated that TA significantly alleviated the swelling of human vaginal epithelial cells and improved cell membrane integrity caused by *P. kudriavzevii* infection. Moreover, TA markedly inhibited the activation of the NLRP3 inflammasome by *P. kudriavzevii*, thereby reducing the expression levels of the pro-inflammatory cytokines IL-18 and IL-1β. This mechanism may be an important pathway through which TA mitigates the inflammatory response in VVC caused by *P. kudriavzevii*. These results indicate that TA can effectively reduce pyroptosis in human vaginal epithelial cells induced by *P. kudriavzevii* via the NLRP3 inflammasome pathway. Notably, this is the first study to elucidate the protective mechanism of tannic acid against *P. kudriavzevii* infection from the perspective of pyroptosis. Although this study primarily focused on the pyroptosis pathway, TA may also regulate other forms of cell death. Based on the existing evidence, we propose that the mechanism by which TA, as a potential antifungal agent, exerts its protective effects through the regulation of pyroptosis warrants further investigation.

This study has three main limitations. First, we did not include fluconazole-susceptible standard strains in the combination experiments. Future studies will therefore explore experimental protocols with lower concentration ranges suitable for highly susceptible strains to comprehensively evaluate the universality of the TA-fluconazole synergistic effect. Second, although the present study evaluated fungal adhesion and early surface colonization using confocal microscopy, quantification of specific extracellular polymeric substance (EPS) components—including exopolysaccharides, extracellular DNA (eDNA), and matrix proteins—was not performed. Consequently, we cannot conclusively demonstrate the presence of structurally mature biofilms. Future studies will employ scanning electron microscopy (SEM) combined with fluorescent labeling techniques (e.g., ConA-FITC for polysaccharides, SYTO 9 for eDNA) to comprehensively characterize EPS composition and validate true biofilm architecture. Finally, this study used the immortalized VK2/E6E7 cell line rather than primary human vaginal epithelial cells. While widely accepted for studying vaginal epithelial cell–*Candida* interactions, it may not fully recapitulate the phenotypic and functional characteristics of primary cells in vivo. Future studies will validate these findings using primary vaginal epithelial cells or ex vivo tissue models to ensure translational relevance.

In summary, our study has confirmed the significant inhibitory effects of TA on *P. kudriavzevii* and its anti-inflammatory protective effects on human vaginal epithelial cells infected with *P. kudriavzevii*. Moreover, TA has been shown to enhance the sensitivity of clinical fluconazole-resistant *C. albicans* and *N. glabrata* to fluconazole. These comparative findings across multiple *Candida* species underscore the potential of TA as a broad-spectrum anti-*Candida* therapeutic strategy for drug-resistant VVC, offering a unified approach to manage the diverse etiology of this common gynecological infection. Therefore, our research suggests that TA may be a promising candidate for the treatment of VVC.

## Figures and Tables

**Table 1:** Antifungal Activity of TA in Combination with Fluconazole against Clinical Fluconazole-Resistant Candida Strains.

Strains	FCZ MIC90	FCZ + 3 μg/mL TA MIC90
*C. albicans* (06-37)	>128	0.25
*C. albicans* (09-07)	>128	0.5
*C. albicans* (C1-17)	>128	0.5
*C. albicans* (C1-19)	>128	0.5
*C. glabrata* (Y10-5)	>128	0.25

The MIC90 (90% Minimal Inhibitory Concentration) is defined as the lowest concentration of an antimicrobial agent required to inhibit the growth of 90% of the tested microbial strains.

## Data Availability

All experimental data supporting the findings are available from the corresponding author upon reasonable request.

## Supplementary Materials

The following supporting information can be downloaded at: https://www.mdpi.com/article/10.3390/pathogens15050464/s1, Supplementary Table S1. Sequence information for qRT-PCR primers.

## References

[1] Doi A.M., Pignatari A.C., Edmond M.B., Marra A.R., Camargo L.F., Siqueira R.A., da Mota V.P., Colombo A.L. (2016). Epidemiology and Microbiologic Characterization of Nosocomial Candidemia from a Brazilian National Surveillance Program. PLoS ONE.

[2] Monzó-Gallo P., Chumbita M., Lopera C., Aiello T.F., Peyrony O., Bodro M., Herrera S., Sempere A., Fernández-Pittol M., Cuesta G. (2023). Real-life epidemiology and current outcomes of hospitalized adults with invasive fungal infections. Med. Mycol..

[3] Fang W., Wu J., Cheng M., Zhu X., Du M., Chen C., Liao W., Zhi K., Pan W. (2023). Diagnosis of invasive fungal infections: Challenges and recent developments. J. Biomed. Sci..

[4] Zhang L., Xiao J., Du M., Lei W., Yang W., Xue X. (2023). Post-translational modifications confer amphotericin B resistance in Candida krusei isolated from a neutropenic patient. Front. Immunol..

[5] Faria D.R., Sakita K.M., Capoci I.R.G., Arita G.S., Rodrigues-Vendramini F.A.V., de Oliveira Junior A.G., Soares Felipe M.S., Bonfim de Mendonça P.S., Svidzinski T.I.E., Kioshima E.S. (2020). Promising antifungal activity of new oxadiazole against Candida krusei. PLoS ONE.

[6] Pfaller M.A., Diekema D.J., Gibbs D.L., Newell V.A., Nagy E., Dobiasova S., Rinaldi M., Barton R., Veselov A. (2008). Candida krusei, a multidrug-resistant opportunistic fungal pathogen: Geographic and temporal trends from the ARTEMIS DISK Antifungal Surveillance Program, 2001 to 2005. J. Clin. Microbiol..

[7] Gómez-Gaviria M., Mora-Montes H.M. (2020). Current Aspects in the Biology, Pathogeny, and Treatment of Candida krusei, a Neglected Fungal Pathogen. Infect. Drug Resist..

[8] Forastiero A., Garcia-Gil V., Rivero-Menendez O., Garcia-Rubio R., Monteiro M.C., Alastruey-Izquierdo A., Jordan R., Agorio I., Mellado E. (2015). Rapid development of Candida krusei echinocandin resistance during caspofungin therapy. Antimicrob. Agents Chemother..

[9] Pappas P.G., Kauffman C.A., Andes D.R., Clancy C.J., Marr K.A., Ostrosky-Zeichner L., Reboli A.C., Schuster M.G., Vazquez J.A., Walsh T.J. (2016). Clinical Practice Guideline for the Management of Candidiasis: 2016 Update by the Infectious Diseases Society of America. Clin. Infect. Dis..

[10] Sobel J.D. (2007). Vulvovaginal candidosis. Lancet.

[11] Ilkit M., Guzel A.B. (2011). The epidemiology, pathogenesis, and diagnosis of vulvovaginal candidosis: A mycological perspective. Crit. Rev. Microbiol..

[12] Singh S., Sobel J.D., Bhargava P., Boikov D., Vazquez J.A. (2002). Vaginitis due to Candida krusei: Epidemiology, clinical aspects, and therapy. Clin. Infect. Dis..

[13] Güzel A.B., Aydın M., Meral M., Kalkancı A., Ilkit M. (2013). Clinical characteristics of Turkish women with Candida krusei vaginitis and antifungal susceptibility of the C. krusei isolates. Infect. Dis. Obstet. Gynecol..

[14] Zhao Y., Nagasaki Y., Kordalewska M., Press E.G., Shields R.K., Nguyen M.H., Clancy C.J., Perlin D.S. (2016). Rapid Detection of FKS-Associated Echinocandin Resistance in Candida glabrata. Antimicrob. Agents Chemother..

[15] Beyda N.D., John J., Kilic A., Alam M.J., Lasco T.M., Garey K.W. (2014). FKS mutant Candida glabrata: Risk factors and outcomes in patients with candidemia. Clin. Infect. Dis..

[16] Xu J., Liu R., Sun F., An L., Shang Z., Kong L., Yang M. (2019). Eucalyptal D Enhances the Antifungal Effect of Fluconazole on Fluconazole-Resistant Candida albicans by Competitively Inhibiting Efflux Pump. Front. Cell. Infect. Microbiol..

[17] Tong Y., Zhang J., Sun N., Wang X.M., Wei Q., Zhang Y., Huang R., Pu Y., Dai H., Ren B. (2021). Berberine reverses multidrug resistance in Candida albicans by hijacking the drug efflux pump Mdr1p. Sci. Bull..

[18] Khanbabaee K., van Ree T. (2001). Tannins: Classification and definition. Nat. Prod. Rep..

[19] Baldwin A., Booth B.W. (2022). Biomedical applications of tannic acid. J. Biomater. Appl..

[20] Youness R.A., Kamel R., A. Elkasabgy N., Shao P., Farag M.A. (2021). Recent Advances in Tannic Acid (Gallotannin) Anticancer Activities and Drug Delivery Systems for Efficacy Improvement; A Comprehensive Review. Molecules.

[21] Jing W., Xiaolan C., Yu C., Feng Q., Haifeng Y. (2022). Pharmacological effects and mechanisms of tannic acid. Biomed. Pharmacother..

[22] Song N., Kan S., Pang Q., Mei H., Zheng H., Li D., Cui F., Lv G., An R., Li P. (2022). A prospective study on vulvovaginal candidiasis: Multicentre molecular epidemiology of pathogenic yeasts in China. J. Eur. Acad. Dermatol. Venereol..

[23] Gauwerky K., Borelli C., Korting H.C. (2009). Targeting virulence: A new paradigm for antifungals. Drug Discov. Today.

[24] Nickerson K.W., Atkin A.L., Hornby J.M. (2006). Quorum sensing in dimorphic fungi: Farnesol and beyond. Appl. Environ. Microbiol..

[25] Niimi M., Firth N.A., Cannon R.D. (2010). Antifungal drug resistance of oral fungi. Odontology.

[26] Mukherjee P.K., Chandra J., Kuhn D.M., Ghannoum M.A. (2003). Mechanism of fluconazole resistance in Candida albicans biofilms: Phase-specific role of efflux pumps and membrane sterols. Infect. Immun..

[27] Glazier V.E. (2022). EFG1, Everyone’s Favorite Gene in Candida albicans: A Comprehensive Literature Review. Front. Cell Infect. Microbiol..

[28] La Bella A.A., Andersen M.J., Gervais N.C., Molina J.J., Molesan A., Stuckey P.V., Wensing L., Nobile C.J., Shapiro R.S., Santiago-Tirado F.H. (2023). The catheterized bladder environment promotes Efg1- and Als1-dependent Candida albicans infection. Sci. Adv..

[29] Zhang K., Sun I.G., Liao B., Yang Y., Ma H., Jiang A., Chen S., Guo Q., Ren B. (2023). Streptococcus mutans sigX-inducing peptide inhibits the virulence of Candida albicans and oral candidiasis through the Ras1-cAMP-Efg1 pathway. Int. J. Antimicrob. Agents.

[30] Martorano-Fernandes L., Goodwine J.S., Ricomini-Filho A.P., Nobile C.J., Del Bel Cury A.A. (2023). Candida albicans Adhesins Als1 and Hwp1 Modulate Interactions with Streptococcus mutans. Microorganisms.

[31] Hosseini S.S., Ghaemi E., Noroozi A., Niknejad F. (2019). Zinc Oxide Nanoparticles Inhibition of Initial Adhesion and ALS1 and ALS3 Gene Expression in Candida albicans Strains from Urinary Tract Infections. Mycopathologia.

[32] Soll D.R. (2008). Candida biofilms: Is adhesion sexy?. Curr. Biol..

[33] Lin L., Wang M., Zeng J., Mao Y., Qin R., Deng J., Ouyang X., Hou X., Sun C., Wang Y. (2023). Sequence Variation of Candida albicans Sap2 Enhances Fungal Pathogenicity via Complement Evasion and Macrophage M2-Like Phenotype Induction. Adv. Sci..

[34] Yano J., Yu A., Fidel P.L., Noverr M.C. (2016). Transcription Factors Efg1 and Bcr1 Regulate Biofilm Formation and Virulence during Candida albicans-Associated Denture Stomatitis. PLoS ONE.

[35] Jones S. (2004). An overview of the basic helix-loop-helix proteins. Genome Biol..

[36] Kramara J., Wakade R.S., Frazer C., Stamnes M.A., Bennett R.J., Krysan D.J. (2025). The Candida albicans transcription factor Efg1 governs hyphal morphogenesis independently of the cAMP-protein kinase A pathway. mBio.

[37] Hoyer L.L., Cota E. (2016). Candida albicans Agglutinin-Like Sequence (Als) Family Vignettes: A Review of Als Protein Structure and Function. Front. Microbiol..

[38] Zhao X., Oh S.H., Coleman D.A., Hoyer L.L. (2022). ALS1 Deletion Increases the Proportion of Small Cells in a Candida albicans Culture Population: Hypothesizing a Novel Role for Als1. Front. Cell. Infect. Microbiol..

[39] Padovan A.C., Chaves G.M., Colombo A.L., Briones M.R. (2009). A novel allele of HWP1, isolated from a clinical strain of Candida albicans with defective hyphal growth and biofilm formation, has deletions of Gln/Pro and Ser/Thr repeats involved in cellular adhesion. Med. Mycol..

[40] Orsi C.F., Borghi E., Colombari B., Neglia R.G., Quaglino D., Ardizzoni A., Morace G., Blasi E. (2014). Impact of Candida albicans hyphal wall protein 1 (HWP1) genotype on biofilm production and fungal susceptibility to microglial cells. Microb. Pathog..

[41] Hirayama T., Miyazaki T., Tanaka R., Kitahori N., Yoshida M., Takeda K., Ide S., Iwanaga N., Tashiro M., Takazono T. (2025). TAC1b mutation in Candida auris decreases manogepix susceptibility owing to increased CDR1 expression. Antimicrob. Agents Chemother..

[42] Shukla S., Rai V., Saini P., Banerjee D., Menon A.K., Prasad R. (2007). Candida drug resistance protein 1, a major multidrug ATP binding cassette transporter of Candida albicans, translocates fluorescent phospholipids in a reconstituted system. Biochemistry.

[43] Kordalewska M., Cancino-Prado G., de Almeida Júnior J.N., Brasil Brandão I., de Souza Peral R.T., Colombo A.L., Perlin D.S. (2023). Novel Non-Hot Spot Modification in Fks1 of Candida auris Confers Echinocandin Resistance. Antimicrob. Agents Chemother..

[44] Daneshnia F., Arastehfar A., Lombardi L., Binder U., Scheler J., Vahedi Shahandashti R., Hagen F., Lass-Flörl C., Mansour M.K., Butler G. (2023). Candida parapsilosis isolates carrying mutations outside FKS1 hotspot regions confer high echinocandin tolerance and facilitate the development of echinocandin resistance. Int. J. Antimicrob. Agents.

[45] Bras G., Bochenska O., Rapala-Kozik M., Guevara-Lora I., Faussner A., Kozik A. (2012). Extracellular aspartic protease SAP2 of Candida albicans yeast cleaves human kininogens and releases proinflammatory peptides, Met-Lys-bradykinin and des-Arg(9)-Met-Lys-bradykinin. Biol. Chem..

[46] Zhou R., Yazdi A.S., Menu P., Tschopp J. (2011). A role for mitochondria in NLRP3 inflammasome activation. Nature.

[47] Dostert C., Pétrilli V., Van Bruggen R., Steele C., Mossman B.T., Tschopp J. (2008). Innate immune activation through Nalp3 inflammasome sensing of asbestos and silica. Science.

[48] Gaidt M.M., Ebert T.S., Chauhan D., Schmidt T., Schmid-Burgk J.L., Rapino F., Robertson A.A., Cooper M.A., Graf T., Hornung V. (2016). Human Monocytes Engage an Alternative Inflammasome Pathway. Immunity.

[49] Schroder K., Tschopp J. (2010). The inflammasomes. Cell.

[50] McKenzie B.A., Dixit V.M., Power C. (2020). Fiery Cell Death: Pyroptosis in the Central Nervous System. Trends Neurosci..

[51] Ishida K., de Mello J.C., Cortez D.A., Filho B.P., Ueda-Nakamura T., Nakamura C.V. (2006). Influence of tannins from Stryphnodendron adstringens on growth and virulence factors of Candida albicans. J. Antimicrob. Chemother..

[52] Morey A.T., de Souza F.C., Santos J.P., Pereira C.A., Cardoso J.D., de Almeida R.S., Costa M.A., de Mello J.C., Nakamura C.V., Pinge-Filho P. (2016). Antifungal Activity of Condensed Tannins from Stryphnodendron adstringens: Effect on Candida tropicalis Growth and Adhesion Properties. Curr. Pharm. Biotechnol..

[53] Latté K.P., Kolodziej H. (2000). Antifungal effects of hydrolysable tannins and related compounds on dermatophytes, mould fungi and yeasts. Z. Naturforsch. Sect. C-A J. Biosci..

[54] Blostein F., Levin-Sparenberg E., Wagner J., Foxman B. (2017). Recurrent vulvovaginal candidiasis. Ann. Epidemiol..

[55] Denning D.W., Kneale M., Sobel J.D., Rautemaa-Richardson R. (2018). Global burden of recurrent vulvovaginal candidiasis: A systematic review. Lancet Infect. Dis..

[56] Bruno V.M., Shetty A.C., Yano J., Fidel P.L., Noverr M.C., Peters B.M. (2015). Transcriptomic analysis of vulvovaginal candidiasis identifies a role for the NLRP3 inflammasome. mBio.

[57] Ardizzoni A., Wheeler R.T., Pericolini E. (2021). It Takes Two to Tango: How a Dysregulation of the Innate Immunity, Coupled with Candida Virulence, Triggers VVC Onset. Front. Microbiol..

[58] Sp N., Kang D.Y., Kim D.H., Yoo J.S., Jo E.S., Rugamba A., Jang K.J., Yang Y.M. (2020). Tannic Acid Inhibits Non-small Cell Lung Cancer (NSCLC) Stemness by Inducing G_0_/G_1_ Cell Cycle Arrest and Intrinsic Apoptosis. Anticancer Res..

[59] Kasiram M.Z., Hapidin H., Abdullah H., Hashim N.M., Azlina A., Sulong S. (2022). Tannic acid enhances cisplatin effect on cell proliferation and apoptosis of human osteosarcoma cell line (U2OS). Pharmacol. Rep..

[60] Bona N.P., Pedra N.S., Azambuja J.H., Soares M.S.P., Spohr L., Gelsleichter N.E., Meine B.d.M., Sekine F.G., Mendonça L.T., de Oliveira F.H. (2020). Tannic acid elicits selective antitumoral activity in vitro and inhibits cancer cell growth in a preclinical model of glioblastoma multiforme. Metab. Brain Dis..

[61] Chen M.C., Annseles Rajula S., Bharath Kumar V., Hsu C.H., Day C.H., Chen R.J., Wang T.F., Viswanadha V.P., Li C.C., Huang C.Y. (2022). Tannic acid attenuate AKT phosphorylation to inhibit UMUC3 bladder cancer cell proliferation. Mol. Cell. Biochem..

[62] Yulak F., Ergul M. (2024). Tannic acid protects neuroblastoma cells against hydrogen peroxide-triggered oxidative stress by suppressing oxidative stress and apoptosis. Brain Res..

[63] Dan Dunn J., Alvarez L.A., Zhang X., Soldati T. (2015). Reactive oxygen species and mitochondria: A nexus of cellular homeostasis. Redox Biol..

[64] Zorov D.B., Juhaszova M., Sollott S.J. (2014). Mitochondrial reactive oxygen species (ROS) and ROS-induced ROS release. Physiol. Rev..

[65] Iyer S.S., He Q., Janczy J.R., Elliott E.I., Zhong Z., Olivier A.K., Sadler J.J., Knepper-Adrian V., Han R., Qiao L. (2013). Mitochondrial cardiolipin is required for Nlrp3 inflammasome activation. Immunity.

[66] Weindel C.G., Ellzey L.M., Martinez E.L., Watson R.O., Patrick K.L. (2023). Gasdermins gone wild: New roles for GSDMs in regulating cellular homeostasis. Trends Cell Biol..

