# Supplementary material for: Exploring the Mechanism of Tannic Acid Against Pichia kudriavzevii in the VK2/E6E7 Vaginal Epithelial Cell Line and Its Synergy with Azoles on Drug-Resistant Candida Species
Source: Pathogens. 2026 Apr 24;15(5):464. doi: 10.3390/pathogens15050464 (PMC13209263; doi:10.3390/pathogens15050464)
Supplement: Supplementary file 1 [file pathogens-15-00464-s001.zip › pathogens-4210395-supplementary.pdf]

Supplementary Table 1. Sequence information for qRT-PCR primers

Gene name	Primer Sequences (5' to 3')
ALS1 Forward	GCAAACCCAGGAGACACATTCAC
ALS1 Reverse	AACACCGTCAGCAGTCAAATCAAC
ALS3 Forward	CTAATGCTGCTACGTATAATT
ALS3 Reverse	CCTGAAATTGACATGTAGCA
CDR1 Forward	GTACTATCCATCAACCATCAGCACTT
CDR1 Reverse	GCCGTTCTTCCACCTTTTTGTA
EFG1 Forward	TATGCCCCAGCAAACAACTG
EFG1 Reverse	TTGTTGTCCTGCTGTCTGTC
EGR11 Forward	GCTGCTGCCAAAGCTAATTC
EGR11 Reverse	TCTATGTCTACCACCACCAAATG
FKS1 Forward	GCCGTACATTTGATGTCATT
FKS1 Reverse	AAAGTACCAGAAGTGACAAC
HWP1 Forward	AGGTAGACGGTCAAGGTGAAACAG
HWP1 Reverse	TGGCTCTTGTGGTTGTTGTTGTGTG
SAP2 Forward	CAAGTGGTTCATCAGCTTCAC
SAP2 Reverse	TTATTTGTCCCGTGGCAG
ACT1 Forward	CCAGCTTTCTACGTTTCC
ACT1 Reverse	CTGTAACCACGTTCAGAC
18S Forward	AATTACCCAATCCCGACAC
18S Reverse	TGCAACAACTTTAATATACGC
